# A comprehensive and high-quality collection of *Escherichia coli* genomes and their genes

**DOI:** 10.1099/mgen.0.000499

**Published:** 2021-01-08

**Authors:** Gal Horesh, Grace A. Blackwell, Gerry Tonkin-Hill, Jukka Corander, Eva Heinz, Nicholas R. Thomson

**Affiliations:** ^1^​ Parasites and Microbes, Wellcome Sanger Institute, Wellcome Genome Campus, Hinxton, Cambridgeshire CB10 1RQ, UK; ^2^​ EMBL-EBI, Wellcome Genome Campus, Hinxton, Cambridgeshire, UK; ^3^​ Department of Biostatistics, University of Oslo, Oslo, Norway; ^4^​ Department of Mathematics and Statistics, Helsinki Institute for Information Technology (HIIT), University of Helsinki, Helsinki, Finland; ^5^​ Department of Vector Biology and Clinical Sciences, Liverpool School of Tropical Medicine, Liverpool L3 5QA, UK; ^6^​ Department of Infectious and Tropical Diseases, London School of Hygiene and Tropical Medicine, London WC1E 7HT, UK

**Keywords:** antimicrobial resistance, *Escherichia coli*, horizontal gene transfer, pan-genome, *Shigella*

## Abstract

*
Escherichia coli* is a highly diverse organism that includes a range of commensal and pathogenic variants found across a range of niches and worldwide. In addition to causing severe intestinal and extraintestinal disease, *E. coli* is considered a priority pathogen due to high levels of observed drug resistance. The diversity in the *E. coli* population is driven by high genome plasticity and a very large gene pool. All these have made *E. coli* one of the most well-studied organisms, as well as a commonly used laboratory strain. Today, there are thousands of sequenced *E. coli* genomes stored in public databases. While data is widely available, accessing the information in order to perform analyses can still be a challenge. Collecting relevant available data requires accessing different sources, where data may be stored in a range of formats, and often requires further manipulation and processing to apply various analyses and extract useful information. In this study, we collated and intensely curated a collection of over 10 000 *E. coli* and *Shigella* genomes to provide a single, uniform, high-quality dataset. *Shigella* were included as they are considered specialized pathovars of *E. coli *. We provide these data in a number of easily accessible formats that can be used as the foundation for future studies addressing the biological differences between *E. coli* lineages and the distribution and flow of genes in the *E. coli* population at a high resolution. The analysis we present emphasizes our lack of understanding of the true diversity of the *E. coli* species, and the biased nature of our current understanding of the genetic diversity of such a key pathogen.

## Significance as a BioResource to the community

As of today, there are more than 140 000 *Escherichia coli* genomes available on public databases. While data are widely available, collating the data and extracting meaningful information from it often requires multiple steps, computational resources and expert knowledge. Here, we collate a high-quality and comprehensive set of over 10 000 *E. coli* genomes, isolated from human hosts, into a set of manageable files that offer an accessible and usable snapshot of the currently available genome data, linked to a minimal data quality standard. The data provided include a detailed synopsis of the main lineages present, including their antimicrobial and virulence profiles, their complete gene content, and all the associated metadata for each genome. This includes a database that enables the user to compare newly sequenced isolates against the assembled genomes. Additionally, we provide a searchable index that allows the user to query any DNA sequence against the assemblies of the collection. This collection paves the path for many future studies, including those investigating the differences between *E. coli* lineages, following the evolution of different genes in the *E. coli* pan-genome and exploring the dynamics of horizontal gene transfer in this important organism.

## Data Summary

1. The complete aggregated metadata of 10 146 high-quality genomes isolated from human hosts (https://doi.org/10.6084/m9.figshare.13270073, File F1).
2. A PopPUNK database that can be used to query any genome and examine its context relative to this collection (deposited in Figshare – https://doi.org/10.6084/m9.figshare.12650834.v1).
3. A BIGSI index of all the genomes that can be used to easily and quickly query the genomes for any DNA sequence of 61 bp or longer (deposited in Figshare – https://doi.org/10.6084/m9.figshare.12666497.v1).
4. Description and complete profiling of the 50 largest lineages that represent the majority of publicly available human-isolated *Escherichia coli* genomes (https://doi.org/10.6084/m9.figshare.13270073, File F2). Phylogenetic trees of representative genomes of these lineages, presented in this paper, are also provided (https://doi.org/ 10.6084/m9.figshare.13270073, files tree_500.nwk and tree_50.nwk).
5. The complete pan-genome of the 50 largest lineages, which includes the following.
  (a) A fasta file containing a single representative sequence of each gene of the gene pool (https://doi.org/10.6084/m9.figshare.13270073, File F3).
  (b) Complete gene presence/absence across all isolates (https://doi.org/10.6084/m9.figshare.13270073, File F4).
  (c) The frequency of each gene within each of the lineages (https://doi.org/10.6084/m9.figshare.13270073, File F5).
  (d) The representative sequences from each lineage for all the genes (https://doi.org/10.6084/m9.figshare.13270073, File F6).

## Introduction

*Escherichia coli* is a globally distributed, highly diverse organism with a very large gene pool [1–3]. While some variants of *E. coli* are found in the guts of healthy individuals, in animals and in the environment, others cause severe intestinal and extraintestinal life-threatening disease [4]. The diversity between *E. coli* strains is driven by high genome plasticity; genes are regularly gained and lost, leading to high variability in gene content between lineages and isolates [2, 5–7]. The combination of these factors, a large gene pool, genome plasticity, global distribution and ubiquity across niches, make *E. coli* an important genetic storehouse for the spread and wider dissemination of genes, including those that confer resistance and virulence. Indeed, *E. coli* has been designated a priority pathogen by the World Health Organization due to its high levels of drug resistance [8]. Therefore, *E. coli* is a highly relevant organism to study in today’s world, with the increasing spread of antimicrobial resistance (AMR), and for understanding the emergence of new, globally disseminated, bacterial pathogens of relevance to human and animal health.

Eight pathogenic variants of *E. coli *, termed ‘pathotypes’, have been defined based on their site of infection and by distinguishing phenotypic and molecular markers [4]. These are broadly divided into diarrhoeagenic pathotypes, which infect the gastrointestinal tract, and extraintestinal variants, termed extraintestinal *E. coli* (ExPECs), which infect other bodily sites, most notably the urinary tract and the blood. The diarrhoeagenic pathotypes include enteropathogenic *E. coli* (EPEC), enterotoxigenic *E. coli* (ETEC), enterohaemorrhagic *E. coli* (EHEC), enteroaggregative *E. coli* (EAEC), enteroinvasive *E. coli* (EIEC), diffusely adherent *E. coli* (DAEC) and adherent invasive *E. coli* (AIEC) [4]. *Shigella* is defined as a separate genus consisting of four different species, *Shigella sonnei *, *Shigella flexneri *, *Shigella boydii* and *Shigella dysenteriae *, for clinical and historical reasons: however, lineages of all *Shigella* species fall within the *E. coli* species phylogeny. Based on molecular definitions, they can be considered diarrhoeagenic *E. coli* [9, 10] with *Shigella* often classified as an EIEC, as they are clinically and diagnostically similar [4]. EPECs, ETECs and *Shigella* are prevalent in the developing world, where they cause fatal diarrhoea among infants and children [11, 12]. ETECs, EAECs and *Shigella* are the most common causes for travellers’ diarrhoea [13]. EHECs are the only diarrhoeagenic *E. coli* that are a cause for concern in developed countries, as their major reservoir is in the gastrointestinal tracts of cattle [14, 15]. EHEC infections cause severe diarrhoea, and complications of an infection can cause haemolytic uraemic syndrome, a life-threatening condition that can lead to kidney failure [4, 14].

The transition from non-pathogenic or non-antimicrobial-resistant variants of *E. coli* to pathogenic or antimicrobial-resistant, is primarily driven by horizontal gene transfer, through the acquisition of virulence factors or resistance genes on plasmids and other mobile genetic elements [4, 16–19]. The availability of thousands of *E. coli* genomes in public databases provides the opportunity to examine the *E. coli* lineages and their gene pool on a scale and resolution that was not previously possible. Here, we collated over 10 000 *E. coli* and *Shigella* isolate genomes, collected from a combination of publications and public databases, and assembled and annotated the entire collection to a high quality. *Shigella* were included as they are phylogenetically part of the *E. coli* species, and are referred to as *E. coli* throughout. We provide all the aggregated associated metadata, a database to query newly sequenced genomes against the assemblies and a searchable index to query a DNA sequence of interest. Additionally, we characterized the most-common lineages present in this dataset, including their resistance and virulence profiles. Finally, we defined the complete gene content of these lineages, enabling many future studies examining the biological differences between the lineages and unravelling routes of gene movement in the population.

## Methods

### Data collection

**Fig. 1.**
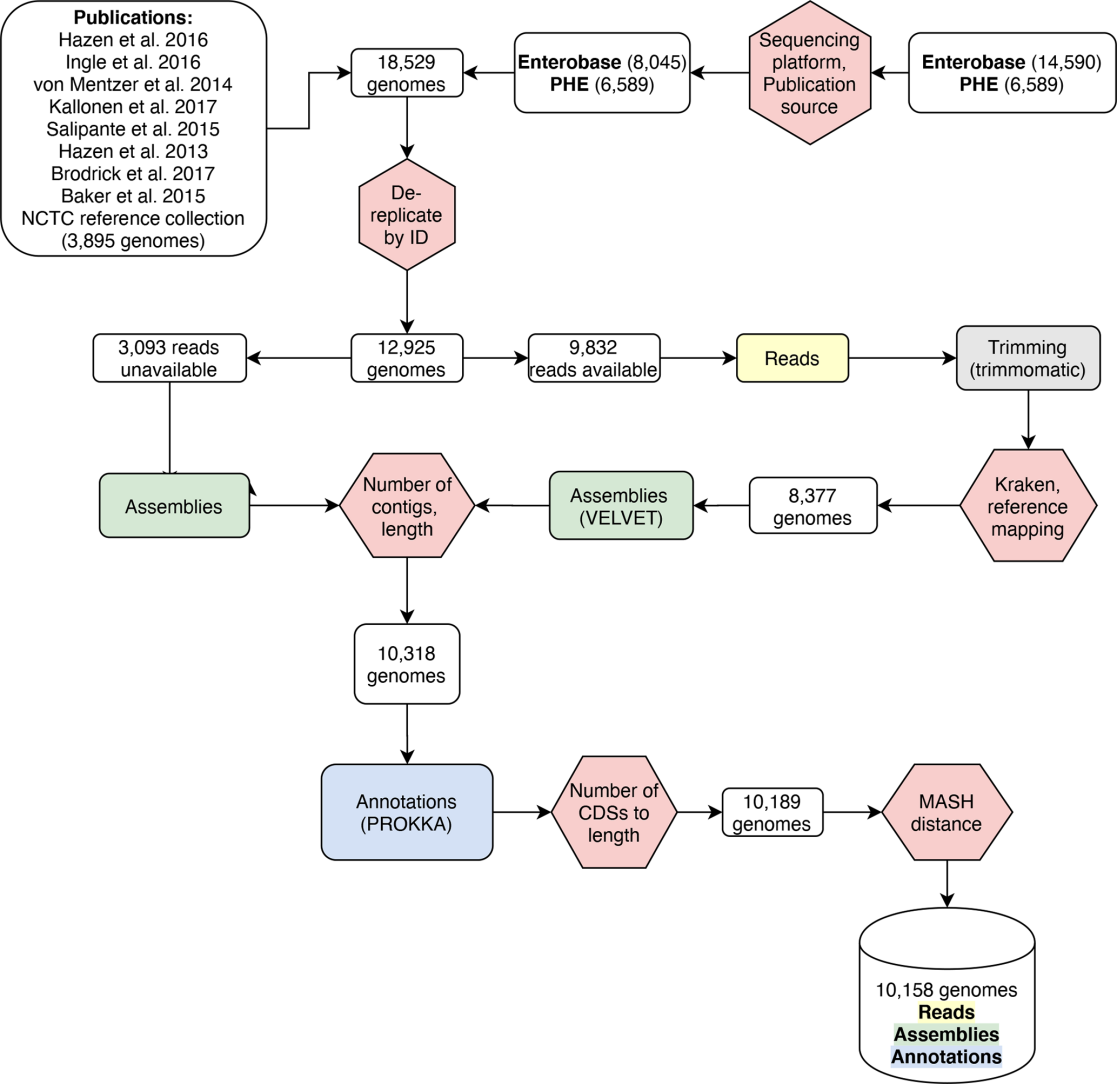
Workflow for constructing the genome collection. Steps taken to obtain a curated, comprehensive and high-quality collection of genomes that includes reads, assemblies and annotation files for each included genome. QC steps are shown in red hexagons, numbers in white rectangles indicate the number of genomes remaining after each QC step. (NCTC: National Collection of Type Cultures.) [20, 21, 22, 23, 24, 25, 26, 70, 71]

A collection of 18 156 *E. coli* (including *Shigella *) genomes, isolated from human hosts, were downloaded and curated to create a final collection of 10 146 genomes, as summarized in Fig. 1. For an initial collection of human *E. coli* genomes for which complete metadata is available, whole-genome sequences were downloaded from the National Center for Biotechnology Information (NCBI) using genome accessions from publications (detailed in File F1 [3, 20–28]). The complete metadata were extracted directly from these publications and these were combined. These genomes were supplemented to include other genomes available from public databases, not associated with publications, for which only partial associated metadata were available. These were predominantly sourced from EnteroBase and from Public Health England (PHE) routine surveillance BioProject (PRJNA315192), downloaded on September 17 2018 [3, 29]. As public read repositories also contain pre-publication data, all publicly available genomes were filtered to include only those for which explicit approval was obtained for use by the submitter.

### Reads

Reads were downloaded from the Sequence Read Archive using fastq-dump (v2.9.2). Reads that had been sequenced by Illumina were trimmed using trimmomatic (v0.33) [30] with the *TruSeq3-PE-2* adaptors, a minimum length of 36 bp, and parameters LEADING=10, TRAILING=10, SLIDING WINDOW=4 : 15 and quality encoding Phred33. When reads were unavailable (3093 genomes), assemblies were shredded into artificial reads using the script available at https://github.com/sanger-pathogens/Fastaq.

Kraken (v0.10.6) was used on the reads to determine what organism had been sequenced [31]. If fewer than 30 % of reads were assigned to *E. coli* or *Shigella* spp., the genome was removed (200 genomes, based on a distribution of these values, Fig. S1, available with the online version of this article). Reads were also mapped to an *E. coli* reference strain cq9 (GCF_003402955.1) and quality-control (QC) statistics were calculated. Samples were removed (1255 genomes) according to the distributions of QC values across all reads (percentage of reads mapped to the reference >60 %, percentage of bases mapped that were mismatches was >0.03, percentage of heterozygous SNPs <3 %; Fig. S1).

### Assembly

Reads were assembled by velvet (v1.2.09) [32] using the prokaryotic assembly pipeline (v2.0.1) with default setting [33]. Assembled genomes were filtered to remove those with more than 600 contigs or those that had a total combined contig length of less than 4 Mb or larger than 6 Mb (1152 genomes, based on a distribution of these values; Fig. S1).

Mash distances were calculated between all the assemblies [34]. Mash uses a minimized database of *k*-mers, i.e. words of size *k*, to represent each genome (based on the MinHash sketch). Mash returns the proportion of shared *k*-mers, the Jaccard distance, between every two genomes as a measure of their genomic distance. A network was constructed so that every genome is represented in a node and two genomes were connected only if their Mash distance was smaller than 0.04 [equivalent to 96 % average nucleotide identity (ANI)] [34]. Isolates from the same species should have an ANI of approximately 95–96 %, i.e. Mash distance smaller than 0.04 [35]. Therefore, genomes were removed (189 genomes) if they were disconnected from the largest connected component, which should represent the *E. coli* and *Shigella* species.

### Coding sequences (CDSs)

Predicted CDSs were predicted using Prokka with a custom training file (v1.5, available at https://doi.org/10.6084/m9.figshare.13270073). Prodigal (v2.6) was trained using a random selected set of 100 genomes from the entire dataset using the ‘prodigal.py’ script available in Panaroo [36, 37]. The training file was used as the input for Prokka to predict the CDSs in the entire dataset. All the genomes were then annotated using the same standardized training properties defined in the training file. There was a linear relationship between the size of the genome and the number of genes called. Genomes that deviated from linear correlation by 500 genes were removed (Fig. S1).

### Constructing the BIGSI index

Each assembly was converted to a non-redundant list of *k*-mers through the construction of De Bruijn graphs (*k*=31) using mccortex v1.0 [38]. All assemblies had between 10^5^ and 10^6^ unique *k*-mers. The parameters chosen for the BIGSI index were h=1 and m=28 000 000, as detailed in the berkeleyDB config file (available at https://doi.org/10.6084/m9.figshare.12666497, file config_10K_00.yaml) and following steps were performed using BIGSI (https://github.com/iqbal-lab-org/BIGSI) [39]. A single hash function (h=1) was applied to each *k*-mer and each assembly was stored as a fixed length (m=28 000 000) Bloom filter (bit-vector). To reduce the overall build time of the index, individual Bloom filters were merged in batches of 500 into matrices using the 'bigsi merge_blooms' command, where the input '--from_files' was a tab separated file where the first column provides the absolute path to the bloom filter and the second is the assembly name. These merged blooms files were then used to build the BIGSI index using 'bigsi large_build' command where the provided 'from_file' input was a file that contains two columns, separated by tab, where the first column details the absolute path to the merged bloom matrices and the second contains all the corresponding assemblies in that merged bloom file, separated by commas. The BIGSI index of the assemblies in this resource, index10k, can be found at https://doi.org/10.6084/m9.figshare.12666497.

### Multilocus sequence typing (MLST)

The sequence type (ST) for each genome was determined by running ‘mlst_check’ (https://github.com/sanger-pathogens/mlst_check) according to the Achtman MLST scheme downloaded from PubMLST on January 22nd 2019 [40]. *Shigella* are included in the Achtman *E. coli* MLST scheme.

### Defining lineages using PopPUNK

PopPUNK (Population Partitioning Using Nucleotide *k*-mers) (v. 1.1.3) was used to group the assemblies into PopPUNK clusters or lineages [41]. PopPUNK uses Mash, a *k*-mer based whole-genome comparison approach, to infer the pairwise core and accessory distances between every two assemblies. The database was constructed with parameters *k*-min=18, *k*-max=30 and step_size=3, as these values produced the correct line fit for estimating the core and accessory distances, as detailed in https://poppunk.readthedocs.io/en/latest/troubleshooting.html#kmer-length. The estimated core and accessory distances between the assemblies were clustered using a two-dimensional Gaussian mixture model (GMM) to identify cut-offs for the within lineage core and accessory distances. The model fitting was applied using six different values of total number of clusters for the GMM (*k*=5, 8, 11, 14, 17 and 20). The scores generated by PopPUNK for all these values were compared. A value of *k*=11 was chosen as it had the overall lowest entropy, i.e. highest confidence in assigning each distance to a cluster, and comparably high overall score. PopPUNK then constructs a network between all assemblies where each node is an assembly, and two assemblies are connected only if their core and accessory distance is below the within lineage core and within lineage accessory distances. All assemblies which are connected to each other in this network are defined as a lineage.

### Phylogenetic analysis

The core-gene phylogeny was inferred from the core-gene alignment generated using Roary for each lineage [42], and a tree from the SNPs in the core-gene alignment, extracted using SNP-sites [43] (v2.3.2), was reconstructed using FastTree [44]. Treemer (v0.3) [45] was used to select ten genomes from each lineage as representatives of that lineage (Table S1). Similarly, Treemer was used to choose a single representative genome from each of the 50 lineages to generate a tree containing only 50 genomes. In both cases, the core-gene phylogeny was inferred from the SNPs of the core-gene alignment generated using Roary on the representative genomes [42]. A maximum-likelihood tree from the informative SNPs, chosen using SNP-sites [43] (v2.3.2), was reconstructed using RAxML (v8.2.8)[46] with 100 bootstrap replicates.

### Phylogroup assignment

ClermonTyping (v1.4.1) was used to assign the *E. coli* phylogroup of the 500 representative *E. coli* genomes [47]. ClermonTyping uses an *in silico* PCR approach of marker genes, following the Clermont phylotyping scheme presented by Clermont *et al*. [48]. This is supplemented by a Mash-based mapping to a curated collection of *E. coli* genomes, for which the phylogroup is known. A lineage was assigned to the phylogroup according to the most common phylogroup assignment of the ten representative strains. The exception was lineage 10, which was assigned to phylogroup D by ClermonTyping as the marker gene *arpA* was not detected in the *in silico* PCR using primer ArpAgpE; however, the assignment did not correspond with the phylogeny and this was corrected to phylogroup E.

### Identification of antimicrobial and virulence genes

A collection of AMR genes was obtained from ResFinder (https://bitbucket.org/genomicepidemiology/resfinder_db/src/master/; downloaded on 06/03/19) [49]. Virulence genes were downloaded from the VirulenceFinder database (https://bitbucket.org/genomicepidemiology/virulencefinder_db/src; downloaded 24/08/18). Read files of genomes (real where available or otherwise artificially generated from the assemblies) were queried for the presence of these known AMR or virulence genes using ariba (v2.14) with default settings [50]. A gene was marked as present only if 80 % of the entry sequence in the database was covered, otherwise it was marked as absent.

### Pathotype assignments

Pathotypes were assigned according to the presence of specific marker virulence genes according to the pathotype-associated markers presented in table 1 in the reference by Robins-Browne *et al*. [51], refined by the source of isolation: if the source of isolation was blood or urine the assignment was ExPEC; if any variant of Shiga-toxin was present the assignment was STEC (Shiga toxin-producing *E. coli *); if *eae* was present the assignment was aEPEC (atypical EPEC)/EPEC; if both Shiga-toxin and *eae* were present the assignment was EHEC; if either *aatA*, *aggR* or *aaiC* were present the assignment was EAEC; if *est* or *elt* were present the assignment was ETEC; if *ipaH9.8* or *ipaD*, characteristic of the invasive virulence plasmid pINV, were present the assignment was EIEC. A pathotype was assigned to a lineage if at least half of the isolates of the lineage were assigned to the same pathotype. *Shigella* lineages were assigned *Shigella* as their pathotype.

### Pan-genome analysis

A pan-genome analysis using Roary [42] was applied on each lineage separately using the default identity cut-off of 0.95, with paralog splitting disabled [42]. The outputs of the pan-genome analysis of each lineage were combined to generate a final collection of gene clusters of the entire dataset in the following steps.

1. Gene cluster definitions, from the Roary analysis within each lineage, were assumed to be the best approximation of the representation of the genes that are well defined within a closely related group of genomes. Note that each gene cluster has multiple members (nucleotide sequences) from that lineage (Fig. S2, step 1). A representative sequence was chosen for each gene cluster as the sequence that had the modal length within that gene cluster. If there was no mode, a sequence with the median length was chosen.
2. A pan-genome analysis using Roary was applied on all lineages in an all-against-all manner using an identity threshold of 0.95 and with paralog splitting disabled, leading to a total of 1081 Roary analyses. This generated gene clusters for each possible lineage pair. Note that, similar to step 1, each gene cluster can have multiple members (nucleotide sequences), but this time from both lineages used in each respective comparison (Fig. S2, step 2).
3. A ‘combined Roary graph’ was constructed, with the gene clusters from the original Roary outputs from step 1 (a Roary analysis on a single lineage only) as nodes (Fig. S2, step 3).
4. Gene cluster of lineage A was connected to a gene cluster of lineage B if there was a gene clustering in their combined Roary analysis (step 2) where (i) 80 % of the members of the gene cluster of A were in the new combined clustering, and (ii) 80 % of the members of the gene cluster of B were also in the combined clustering (Fig. S2, step 4).
5. Following corrections, the connected components of the combined Roary graph were the final set of gene clusters in the entire dataset (Fig. S2, step 6).

The following corrections were applied to add or remove connections between gene clusters in the combined Roary graph (Fig. S2, step 5).

(a) Density-based clustering groups data points based on their density in space, while assuming that data points that belong to the same group are in a region of a high density and are separated from another group by a region of low density. The distance metric used for density-based clustering was the proportion of shared edges (Jaccard index) between every two nodes in the combined Roary graph. This identified spurious connections between genes that were not supported by most pairwise Roary analyses (Fig. S2). This was applied using the ‘dbscan’ method of the Python package sci-kit learn [52 ] with parameters epsilon=0.5 and min_samples=6. Connections between a gene cluster of lineage A and a gene cluster of lineage B that did not belong to the same dbscan cluster were removed.

(b) To correct for under-splitting, all representative nucleotide sequences of each gene cluster of the combined Roary graph were aligned to each other using mafft (v7.310) [53] with default settings. If the alignment of two sequences showed more than 20 % mismatches along the length of the longer sequence, the connection between them in the combined Roary graph was removed (see Fig. S2, step 5 b).

(c) To correct for over-splitting, the representative protein sequences of all the gene clusters of the original Roary outputs were aligned to each other using blastp (version 2.9). Representative sequences which were more than 95 % identical over 80 % of their length were merged (See Fig. S2, step 5 c).

1. Following corrections, the connected components of the combined Roary graph were the final set of gene clusters in the entire dataset (Fig. S2, step 6).

File F6, available at https://doi.org/10.6084/m9.figshare.13270073, contains the representative sequences from the original Roary outputs (step 1) for each gene in the final gene clusters (step 6).

### Statistical analysis

Statistical analyses were performed in R (v3.3+). Ape (v5.3) [54] and ggtree (v1.16.6) [55] were used for phylogenetic analysis and visualization. The ggplot2 (v3.2.1) package was used for plotting [56]. All scripts used in the analysis are available at https://github.com/ghoresh11/ecoli_genome_collection.

## Results

### *E. coli* genomes

A total of 18  156 *E. coli* genomes, isolated from human hosts, were collected from a variety of sources and required multiple processing steps, which are detailed in Methods and summarized in Fig. 1. *Shigella *, which are phylogenetically part of the *E. coli* species, were also included and are referred to as *E. coli* throughout. In short, genome identifiers from publications where complete metadata were available were collected, and combined with identifiers of genomic data from public databases for which only limited metadata were available. Genomes were downloaded, assembled and their CDSs were predicted and annotated. Importantly, to ensure the accuracy of the data, multiple QC measures were applied, reducing the initial dataset and thereby ensuring a final collection of high-quality genomes (Fig. 1). Only genomes for which we received explicit approval for them to be used by the submitter were kept, removing any doubts regarding the ability to use this data for high-resolution analyses. The curated high-quality final genome collection comprises 10 146 genomes on which all the subsequent analysis was performed. This makes this dataset unique as it can be used as a reliable, well-described and curated reference for the diversity of the majority of publicly available human-isolated *E. coli* genomes.

The vast majority of available *E. coli* genomes are from developed countries, collected in surveillance in clinical settings. The clinical samples are mostly generated by agencies that conduct regular investigations of *E. coli* isolates in outbreaks and routine surveillance programmes. These include PHE (5207 genomes), the Food and Drug Administration (FDA) (883 genomes), and the Centers for Disease Control and Prevention (CDC) (561 genomes) (Fig. S3). This explains the bias in the available genomic data with 70 and 15 % of the original samples originating from the UK or the USA, respectively. The remaining genomes originated mostly from other countries in Europe, with only a small fraction of genomes being currently available from Asia, Africa, South America or Oceania.

A total of 38 % of the samples considered here were taken from faeces, blood and urine. The remaining samples were recorded as being from unknown or other human sources (File F1). Isolates from Africa and Asia were exclusively from faecal samples, whereas isolates from Europe and North America included those causing both intestinal and extraintestinal disease (Fig. S3). Where available, the pathotype description was as described in the original publication. Within these isolates, the representation of diarrhoeal-disease-causing *E. coli* pathotypes, EPECs and ETECs, was very low with only 3 and 2 % of the genomes belonging to these pathotypes, respectively.

### Six STs represent more than 50 % of the genomes in the collection

MLST is based on the variation of seven housekeeping genes, the combination of which define a ST. A total of 993 different known STs were identified in the collection. A total of 87 STs (9 %) alone accounted for 80 % of the isolates (Fig. S4). Six STs, 11, 131, 73, 10, 95 and 21, accounted for 50 % of the isolates included here. A total of 790 STs (~80 % of the STs) were represented by five isolates or fewer. Many of the former represent important STs linked to human disease. For instance, ST11 (30 % of all genomes) is associated with EHEC serotype O157:H7, a major foodborne pathogen that can be contracted by eating contaminated foods, specifically beef products, as it lives in the colon of cattle and is an important cause of haemolytic uraemic syndrome in humans [14]. The collection also includes STs of non-O157 EHECs, including STs 17 (2  %) and 21 (2  %). STs 131 (8  %), 73 (4  %) and 95 (3  %) are all STs known to be associated with extraintestinal disease [20, 21, 57]. ST10 (3 %) is a broad-host-range ST, isolates of which have been observed in multiple host species, and include all known *E. coli* pathotypes [58].

### The dataset can be divided into lineages of closely related isolates

As *E. coli* is a highly diverse organism, relying on MLST for subtyping can lead to new ST definitions within a group of closely related isolates due to variation in one of these genes, or otherwise to connections between unrelated isolates due to recombination. Therefore, we grouped the genomes into lineages of closely related isolates using a whole-genome-based approach. PopPUNK extracts and compares words of size *k*, named *k*-mers, from whole genomes to measure the deviation in core-gene sequence termed as the core distance, and the deviation in gene content, termed as the accessory distance, between two genomes [41]. In *E. coli *, the core distance, as estimated by PopPUNK, correlates with the pairwise SNP distance between all the core genes of the two genomes being compared, and the accessory distance correlates with the proportion of shared accessory genes between every two genomes (the Jaccard distance) [41]. Genomes that had both low core and accessory distances were considered to be in the same PopPUNK cluster, defined here as a lineage, as they were highly similar in both their core and accessory genomes.

Based on the rules described above, this grouping produced 1154 lineages. As expected, the distribution of lineage sizes was similar to that defined by MLST with a few large lineages representing most of the population (Fig. S4). A single lineage, lineage 1, contained 34 % of all genomes (File F2). This lineage was mostly comprised of ST11, i.e. O157:H7 EHEC. Similarly, lineage 2 contained 8 % of all genomes and consisted mostly of ST131, a global multidrug-resistant (MDR) ExPEC lineage. The third largest lineage, lineage 3, contained 5 % of all genomes and mostly consisted of isolates belonging to ST73 (File F2).

### Fifty PopPUNK lineages represent more than 75 % of the genomes, and are representative of the currently known *E. coli* population structure

**Fig. 2.**
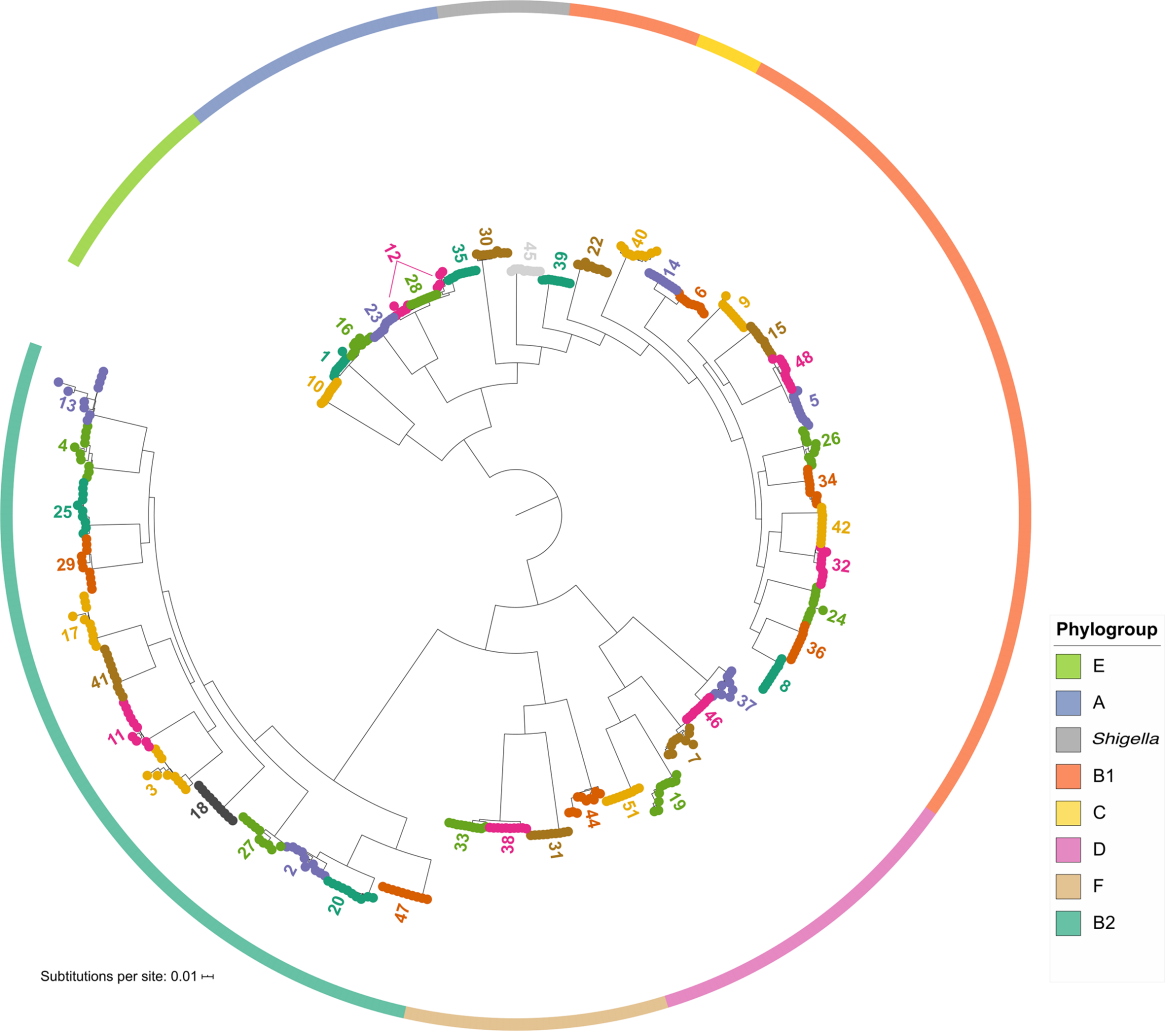
Population structure of the lineages. Core-gene phylogeny of 10 representatives from each of the 50 largest PopPUNK lineages, selected using Treemer [45 ]. The solid coloured outer ring indicates the phylogroup assignment of the representatives of that lineage. The tree was plotted using iTOL [72]. Colours on the tips are used to distinguish between the PopPUNK lineages.

We focused the further analysis of the dataset on the 50 lineages that had at least 20 isolates. Together these lineages included 7693 genomes (76 % of the collection) and 271 different STs (27 % of those described by this collection). To examine the population structure and diversity of the 50 largest lineages, the phylogeny was reconstructed by selecting ten genomes from each lineage that captured most of the diversity of that lineage (see Methods; Table S1), leading to a total of 500 genomes representing the dataset. Their core genome was extracted and the phylogenetic tree from the core-gene alignment was inferred. The phylogenetic analysis confirmed that PopPUNK separated the genomes into clearly distinct lineages based on their core genome (Fig. 2). The exception to this was lineage 12, which was split into two closely related groups. One group was more closely related to lineage 28, whereas the other was closer to lineage 35. The core and accessory distances estimated by PopPUNK showed that indeed, the core distance between PopPUNK clusters 12, 28 and 35 was low; however, they sufficiently deviated in their accessory gene content to be defined as three distinct PopPUNK lineages.

Population genetics studies on *E. coli* have defined the existence of eight deep-branching phylogenetic groups, termed ‘phylogroups’ (A, B1, B2, D, E, F, C and G) [59–62]. While the collection assembled here is biased towards particular STs and we only included lineages with 20 genomes or more, it is evident from Fig. 2 that the collection of genomes spans all *E. coli* phylogroups [18 from B1, 13 from B2, 4 from A, 5 from D, 4 from F, 3 from E, 1 from C, and 2 of *Shigella* representing *S. sonnei* (45) and *S. flexneri* (30)] and, therefore, is representative of the known species diversity [48, 63].

### Associated metadata shows a consistent source of isolation per lineage

The lineages broadly divide into those enriched for isolates collected from faecal samples, and those collected from blood and urine samples (see File F2, Fig. S5). Only lineages 26, 34 and 48 of the intestinal isolate lineages were enriched for samples collected from Africa and Asia. These lineages mostly represented EPEC and ETEC isolates that had been collected from faecal samples in developing countries as part of the the Global Enteric Multicenter Study (GEMS) collection, in contrast to the other lineages containing faecal samples that include STECs or EHECs that had been collected in the high-income settings [12]. Lineage 12, which consisted of 78 % isolates from ST10, was the only lineage that spanned all continents and consisted of all sample types (faecal, blood, urine or unknown).

Where sampling date was available, 39 % of the genomes in the collection were collected in the last 10 years. A number of lineages included older, historically important isolates from the Murray collection [22] (Fig. S5). Notably, lineage 30, which contains *S. flexneri* isolates, had a higher proportion of isolates collected before 1980 relative to the rest of the collection (Wilcox summed rank test, *P* <0.05, Bonferroni corrected).

### Lineages vary substantially in their genome size

The number of genes in a single isolate and the size of the genome varied significantly between the lineages (Fig. S6). The weighted-mean number of genes across all lineages was 4869 genes and the weighted-mean genome length was 5.2 Mbp. Isolates from the *Shigella* lineages 30 and 45 had the smallest genomes, with a genome size of only 4.3 and 4.7 Mbp. Lineages 12, 40 and 48 had the second smallest genome lengths with a mean genome length of ~4.85 Mbp. However, lineages 5, 6, 8, 15 and 48, all from phylogroup B1, had a mean of over 5100 genes per isolate (200 genes more than the dataset mean). The number of predicted genes and genome size were affected by the phylogroup. Lineages in phylogroups E, F and B1 tended to have larger genomes with a few exceptions. Lineages from phylogroup C, B2 and A tended to have smaller genomes. Phylogroup D had a wider range of observed genome sizes.

### Multidrug resistance was predicted for more than half of the isolates in 16 of 50 lineages

**Fig. 3.**
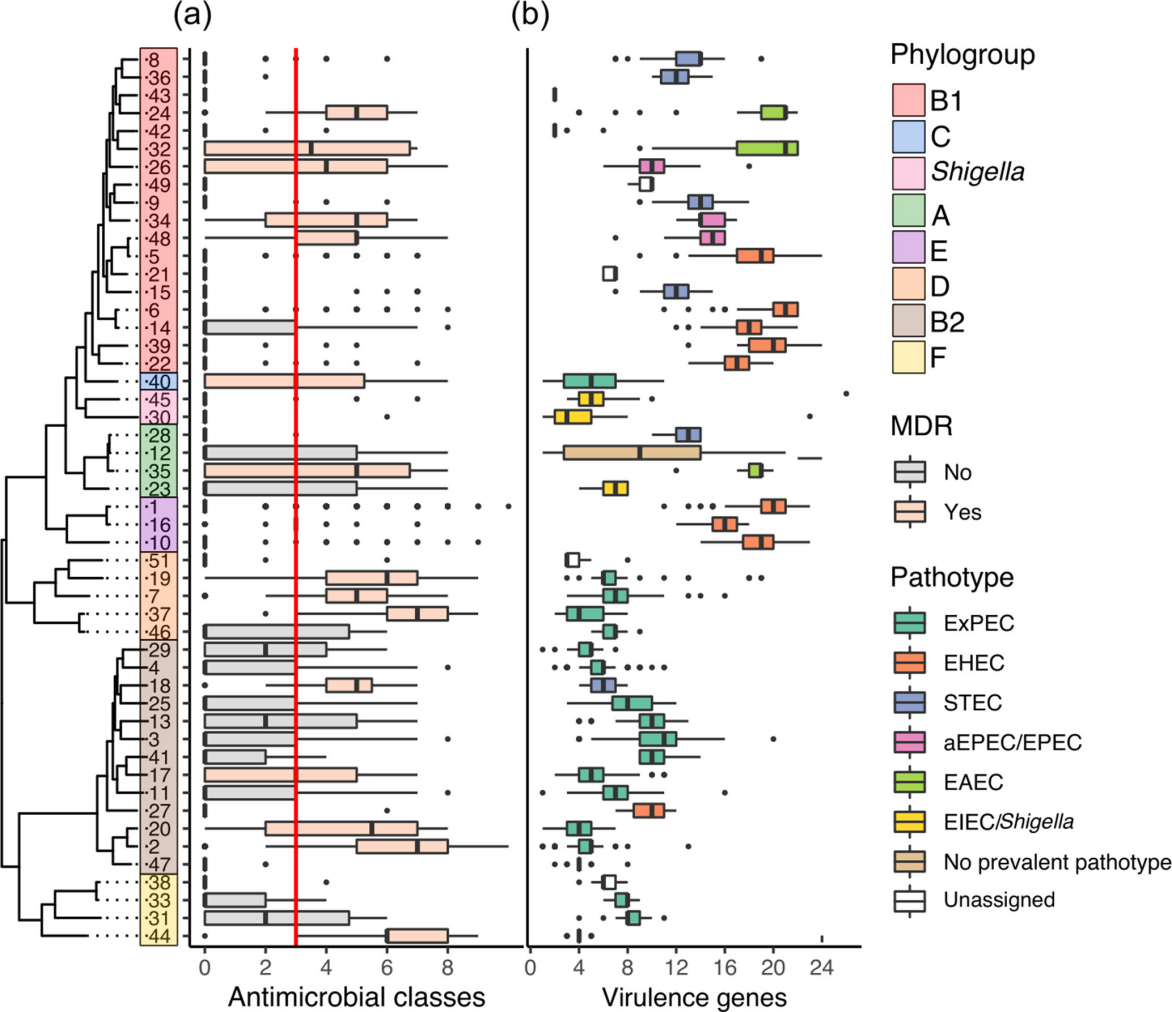
AMR and virulence profiles of the lineages. (a) Number of predicted antimicrobial classes each isolate is resistant to, based on genetic profile by lineage. The red line indicates the threshold for multidrug resistance (predicted resistance to three classes of antimicrobials or more). (b) Number of virulence genes per isolate, by lineage and coloured by the most prevalent predicted pathotype in the lineage. nd, Not determined.

A total of 153 known resistance gene alleles were identified in the collection. The number of known resistance genes within each isolate ranged from none to a maximum of 18 in a single isolate, predicted to confer resistance to up to ten different antimicrobial classes (Fig. 3a, File F1).

Multidrug resistance in an isolate has been defined as resistance to three classes of antibiotics or more [64]. All but five lineages (lineages 21, 36, 43, 47 and 49) had at least one isolate that was MDR. We defined an MDR lineage as a lineage where half of the isolates or more were MDR. A total of 16 of the 50 lineages investigated were MDR (Fig. 3a, File F2). Importantly, this metric is affected by the sampling bias; lineages are MDR because isolates with clinical significance are being sequenced, and it does not inform on the true diversity of AMR genotypes within these lineages in the *E. coli* population. Indeed, *E. coli* isolated from humans have been shown to possess more resistance genes [65]. Half of these lineages were isolated predominantly from blood and urine samples, i.e. ExPECs (lineages 2, 20, 44, 40, 17, 7, 37 and 9). These included lineages 2 and 20, which contain isolates of the global ExPEC lineage ST131. Three of the ExPEC MDR lineages belonged to phylogroup D (lineages 19, 7 and 37). Three other MDR lineages predominantly contained EPEC isolates from the GEMS collection (lineages 26, 34 and 48) [12, 23]. The source of isolation of the remaining five lineages (lineages 32, 35, 18, 16 and 24) was predominantly unknown (Fig. S5).

### Forty-three of fifty lineages are dominated by a single *E. coli* pathotype

Consistent with the collection of *E. coli* isolates being from human hosts and mostly from clinical samples, 439 known virulence genes were observed in our dataset. The isolates had a median of 9 known virulence genes in a single genome, with a maximum value of 26 virulence genes present in a single isolate.

A combination of the source of isolation as well as the detection of a set of marker virulence genes were used to find the most prevalent predicted pathotype within each lineage (see Methods). A total of 44 of 50 lineages were identified as predominantly containing one of the *E. coli* pathotypes, i.e. at least half of the isolates of the lineages were predicted to belong to one of the pathotypes (Fig. 3b). Lineage 12, which mostly consists of *E. coli* isolates typing as ST10, was the only lineage that contained isolates assigned to multiple different pathotypes with no single dominant pathotype (11 % ExPEC, 29 % EAEC, 24 % EPEC, 9 % STEC, 2 % EHEC, 1 % ETEC and 24 % unassigned). The remaining six lineages that were not assigned an *E. coli* pathotype, predominantly from B1 (21, 42, 43, 49 B1; 38, F; and 51, D), had relatively few virulence genes, as well as few AMR genes.

Of the isolates included here, phylogroups B2, F and D predominantly contained ExPEC isolates. Lineages 27 and 18 were the only lineages in phylogroup B2 that contained 67 % EHEC isolates and 33 % aEPEC/EPECs (lineage 27) and 100 % STEC isolates (lineage 18) (Fig. 3b). All phylogroup E lineages contained predominantly EHEC isolates. Phylogroups A and B1 had more diversity of pathotypes, containing lineages that were assigned to the range of diarrhoeagenic pathotypes (EPEC, EHEC, EAEC and EIEC). Lineage 24 of phylogroup B1 contained 38 % isolates that were *stx* and *eae* positive. These are isolates of *E. coli* serotype O104:H4 taken from the 2011 German outbreak, which were classified as the convergence of an EHEC and an EAEC [66]. Lineage 40 was the only ExPEC lineage within the B1-C-A clade.

### Final pan-genome includes a total of 55 039 genes

In order to define the gene content of this reference collection, an initial pan-genome analysis was applied to the lineages separately (see Methods), revealing a low gene diversity within lineages 21, 43 and 49 (Fig. S7). Therefore, these were not included in the detailed description of the pan-genome of the lineages as the low diversity was linked to these being collected at the same time by the FDA. The outputs of the 47 pan-genome analyses of the remaining lineages were combined in order to provide a description of the gene pool in the entire dataset (see Methods). Briefly, a pairwise pan-genome analysis was applied on all CDSs of every two lineages. The grouping of CDSs in every pairwise pan-genome analysis was examined to determine whether two CDSs from two lineages should be labelled as the same gene in the complete dataset.

**Fig. 4.**
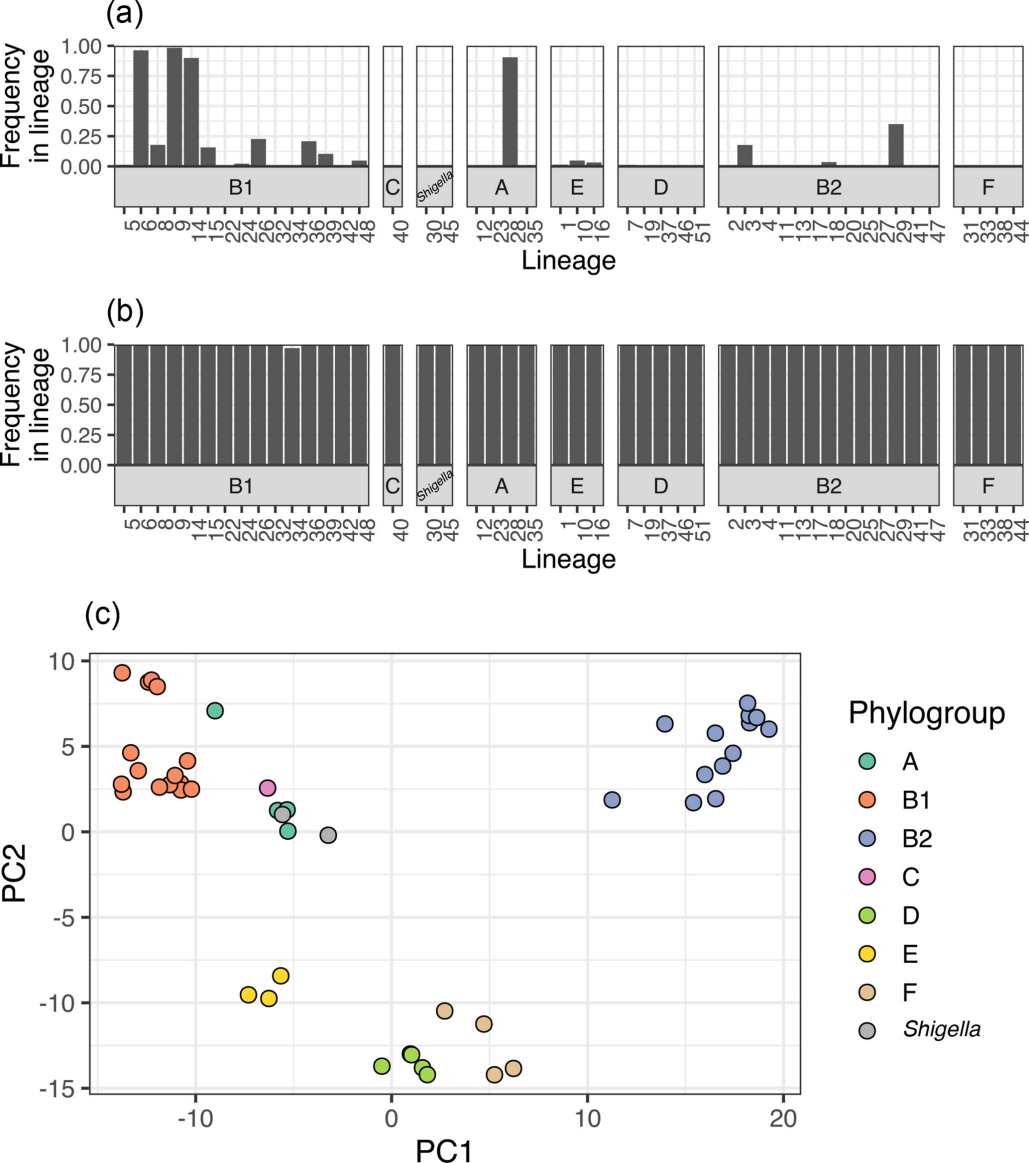
Gene frequencies across the lineages. (a, b) Examples of the frequencies of two genes across the 47 lineages, stratified by phylogroup. (a) The *intA* is present in some lineages and is observed in different frequencies across these. (b) *wzyE* is a core gene observed in a high frequency across all lineages. (c) Principal component analysis plot of the gene frequencies across all lineages, coloured by phylogroup. PC, Principal component.

A total of 55 039 predicted CDSs were identified in this dataset (Files F3–F6). As there are 47 lineages, and a varying number of isolates per lineage, each gene has a frequency within each of the 47 lineages (provided in File F5). For instance, the *intA* gene, encoding a prophage integrase, was observed in 20 of the lineages (Fig. 4a). In two lineages (6 and 9), it was present in over 95 % of isolates, in another eight lineages it was present in intermediate frequencies (between 15 and 95 %) and in the final ten lineages it was present in fewer than 15 % of isolates. In contrast, the gene *wzyE*, a gene involved in antigen biosynthesis, is a core gene that was observed across all lineages in a frequency of over 95 % (Fig. 4b). Principal component analysis on all the gene frequencies across the lineages showed that the first and second principal components explained 17.93 and 7.49 % of the variance and separated the lineages by phylogroup (Fig. 4c).

### Example usage

#### Searching for any DNA sequence in the collection using BIGSI

BIGSI uses a *k*-mer based approach to query any DNA sequence of 61 bp or greater against all the assemblies of the collection [39]. This can be achieved as follows, using the files provided at doi.org/10.6084/m9.figshare.12666497:

*bigsi search -c config_10K_00.yaml -t 0.8*

*ATGAAAAACACAATACATATCAACTTCGCTATTTTTTTAATAATTGCAAATATTATCTACA*

– where config_10K_00.yaml provides the config file to the BIGSI index of the assemblies, and 0.8 is the threshold in *k*-mer similarity (equivalent to 2 mismatches per 100 bps) used to define a match, and ‘ATGAAAAACACAATACATATCAACTTCGCTATTTTTTTAATAATTGCAAATATTATCTACA’ is the sequence being used to search the dataset, compiled here using BIGSI. BIGSI will return all the genome identifiers in the collection that have this sequence in at least 80 % *k*-mer similarity. The properties of these genomes can be investigated in File F1. The user will need to ensure the path to the index is correct (‘filename:’) in the config_10K_00.yaml file. Please refer to the BIGSI documentation (https://github.com/iqbal-lab-org/BIGSI) for full details.

#### Examining the membership of newly sequenced genomes to the lineages in this collection

Newly sequenced genomes can be compared to the lineages in this collection by using the PopPUNK Database provided at doi.org/10.6084/m9.figshare.12650834, as follows:

*poppunk --assign-query --ref-db ecoli_poppunk_db --q-files list_of_genomes.txt --output out*

– where ecoli_poppunk_db is the PopPUNK database provided above, and list_of_genomes.txt is a file containing the list of the new user provided assemblies being queried.

A new directory named ‘out’ is automatically created. The file out_clusters.csv will capture the assignment of each assembly to the lineages defined in the PopPUNK database. The properties of these lineages can be examined in files associated with this article, F1 and F2. Please refer to the PopPUNK documentation (https://poppunk.readthedocs.io/en/latest/) for full details.

#### Examining the distribution of a gene across the species phylogeny

**Fig. 5.**
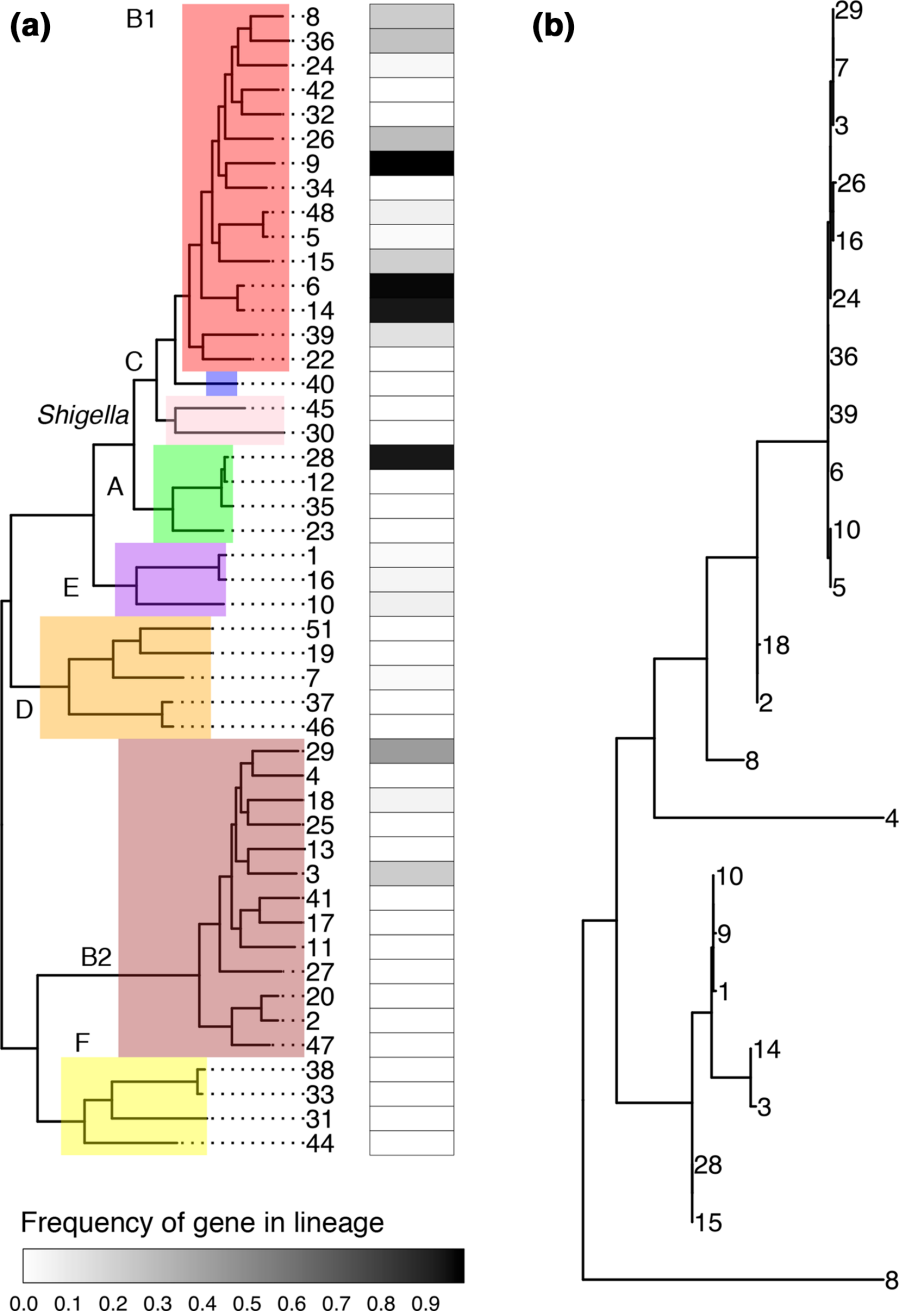
Example usage of the pan-genome to examine the distribution of a single prophage integrase gene (intA_1). (a) The distribution of a gene can be examined across the species tree using the gene frequencies from File F5. The heatmap indicates the fraction of isolates of a lineage that possess the gene. (b) Phylogenetic tree of the gene sequences from each lineage. Sequences of the gene from (a) in each lineage can be extracted from File F6 to examine the species-wide evolution of the gene. Numbers on branch tips indicate the lineage.

A gene of interest can be identified in the pan-genome presented by using alignment tools like blast+ [67] or diamond [68] against the pan-genome reference file provided (File F3). The distribution of the gene named ‘intA_1’, a prophage integrase, in this genome collection can be plotted across the phylogeny of the 47 lineages using the frequencies from the provided File F5 (Fig. 5a). The phylogeny of the specific sequences of each lineage can be drawn using the sequences provided in File F6 (Fig. 5b).

## Discussion

We have created a high-quality, extensively curated dataset of over 10 000 *E. coli* and *Shigella* genomes, linked this to resources that enable this dataset to be queried as a single dataset, and have provided several usage examples. Additionally, we have described in detail the properties of the main lineages present in the collection and their gene (predicted CDS) content. We hope that the data provided in this article will make future studies on *E. coli* more accessible to a wider audience, and will facilitate the investigation of some of the pressing questions in *E. coli* genetics and evolution.

Aggregating data from diverse sources along with their associated metadata is not trivial but, given the increasing number of data sources and data types, essential. Genome identifiers and data formats across publications and databases do not always match, leading to many conversions that are error prone and require knowledge of programming. In addition, computational resources are required in order to apply thousands of assembly and annotation calculations. These are all limiting factors to research. This emphasizes the need to build new resources that maintain high-quality genome collections where users would more easily be able to both retrieve and apply analyses on large collections. Without such resources, information is widely available, but it is practically only usable for a small proportion of scientists with large resources and computational expertise. EnteroBase is a valuable resource that overcomes data accessibility issues by integrating, assembling and analysing the genomic data of specific enteric pathogens from the Sequence Read Archive, while providing researchers with relevant metadata and software [3]. However, as metadata is often associated with a publication, and is not directly linked to the database from which the genome was downloaded, this information is often missing. Even more, describing the gene content by comparing whole-genomic datasets is a much harder problem, which cannot realistically be provided in a high quality in an automated manner across increasing dataset sizes. Therefore, studies on *E. coli* in recent years have either been detailed and focused only on a single pathotype [20, 23–25] or, when utilizing a very large number of genomes, the analyses were limited in their resolution due to the complexity of extracting the information from such large collections [3, 69]. Taken together, the collection presented here represents a detailed, high-quality and accessible dataset that will enable researchers to apply comprehensive comparisons in future investigations on *E. coli *. This includes the PopPUNK and BIGSI databases, which can be used to query newly sequenced isolates or DNA sequences of interest and examine their diversity relative to this collection.

The analysis presented in this paper emphasizes our lack of knowledge on the true diversity of this important species, and that we should redirect our efforts towards sampling to understand the diversity which has yet to be studied. The collection we obtained is biased towards *E. coli* lineages that have clinical significance. The vast majority of genomes were available from Europe and North America, such that the pathotypes comprising the dataset are those that predominantly affect these areas. Of 1154 lineages, there were only 50 that contained at least 20 isolates that were used for defining the gene content. Sampling should be increased in a directed manner in under-represented areas of the world, as well as sampling of non-clinical isolates. Using the PopPUNK database provided in this study, future studies can incorporate new genomes to the dataset provided here and compare *E. coli* isolates from other geographical locations, animals or the environment to the genomes presented here. The PopPUNK database could be expanded and updated in future versions that include these more targeted samples which expand on the diversity presented here.

Biological differences between the lineages were already revealed from the initial descriptions of the lineages presented in this study. There were clear differences in the genome size between the phylogroups and lineages. Higher variability in genome size within a phylogroup or lineage could be an indication of higher rates of gene gain and loss within that lineage. A larger genome size may also help to equip a lineage to survive in a range of niches. These results indicate the importance of this dataset in addressing some important questions regarding the differences between different *E. coli* lineages and gene flow in the *E. coli* population.

## Supplementary Data

Supplementary material 1

Supplementary material 2
