# Supplementary material for: A comprehensive and high-quality collection of Escherichia coli genomes and their genes
Source: Microb Genom. 2021 Jan 8;7(2):000499. doi: 10.1099/mgen.0.000499 (PMC8208696; doi:10.1099/mgen.0.000499)
Supplement: Supplementary material 1 [file mgen-7-499-s001.pdf]

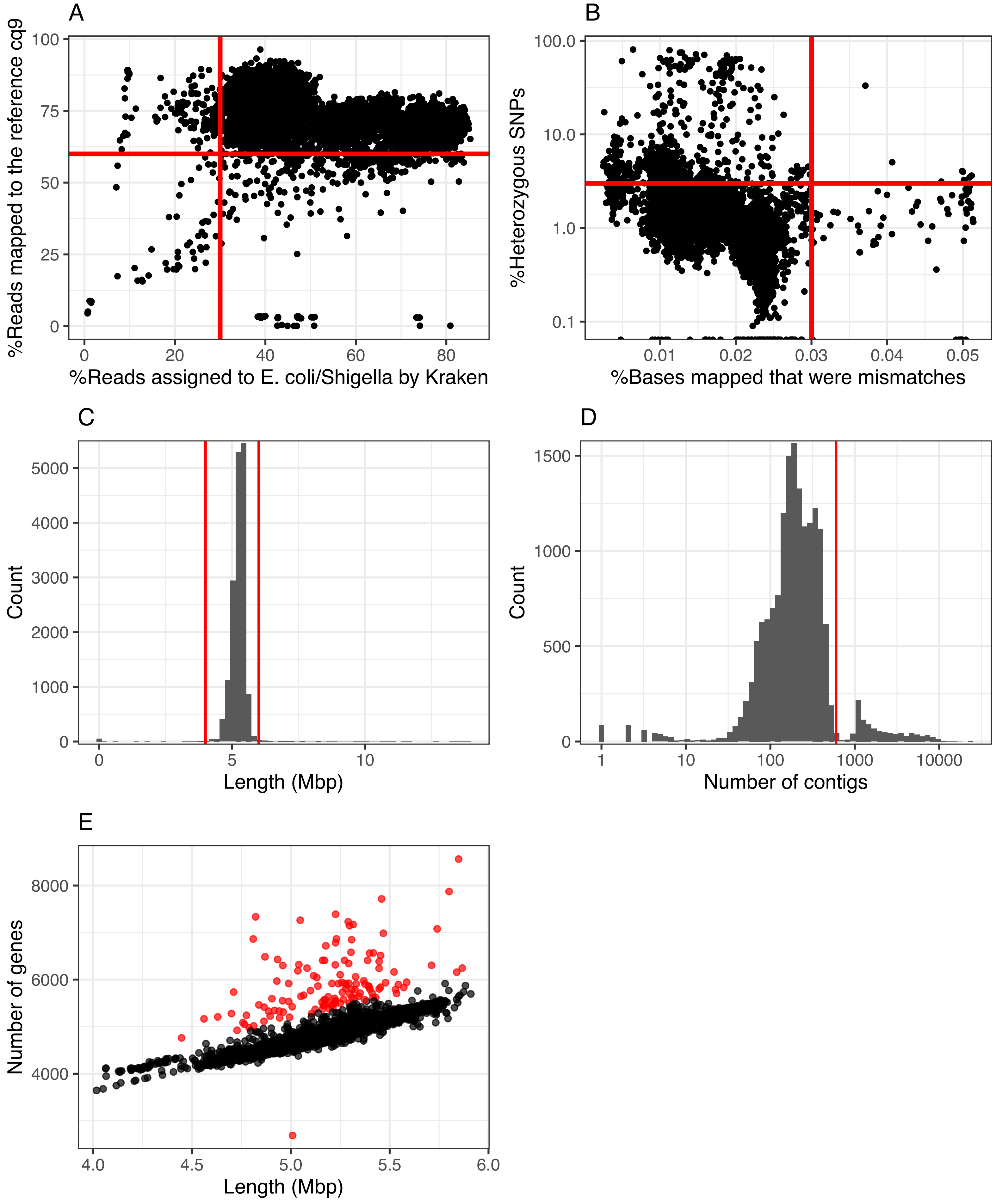

S1: Quality control measures used to filter genomes.A Percentage of reads which were assigned to *E. coli/Shigella* using Kraken relative to the number of reads mapped to an *E. coli* reference cq9. Red lines indicate cut-offs applied, top right corner are all remaining genomes. **B** Percentage of bases mapped which were mismatches relative to the percentage of heterozygous SNPs for each genome. Red lines indicate cut-offs applied, bottom left corner are all remaining genomes. **C** Distribution of genome lengths in the collection. Red lines: genomes shorter than 4 Mb or longer than 6 Mb were removed. **D** Distribution of number of contigs per genome in the collection. Red line: genomes with more than 600 contigs were removed. **E** Correlation between genome length and number of predicted CDSs using Prokka. Red: Genomes which deviated from the expected number of genes were removed.

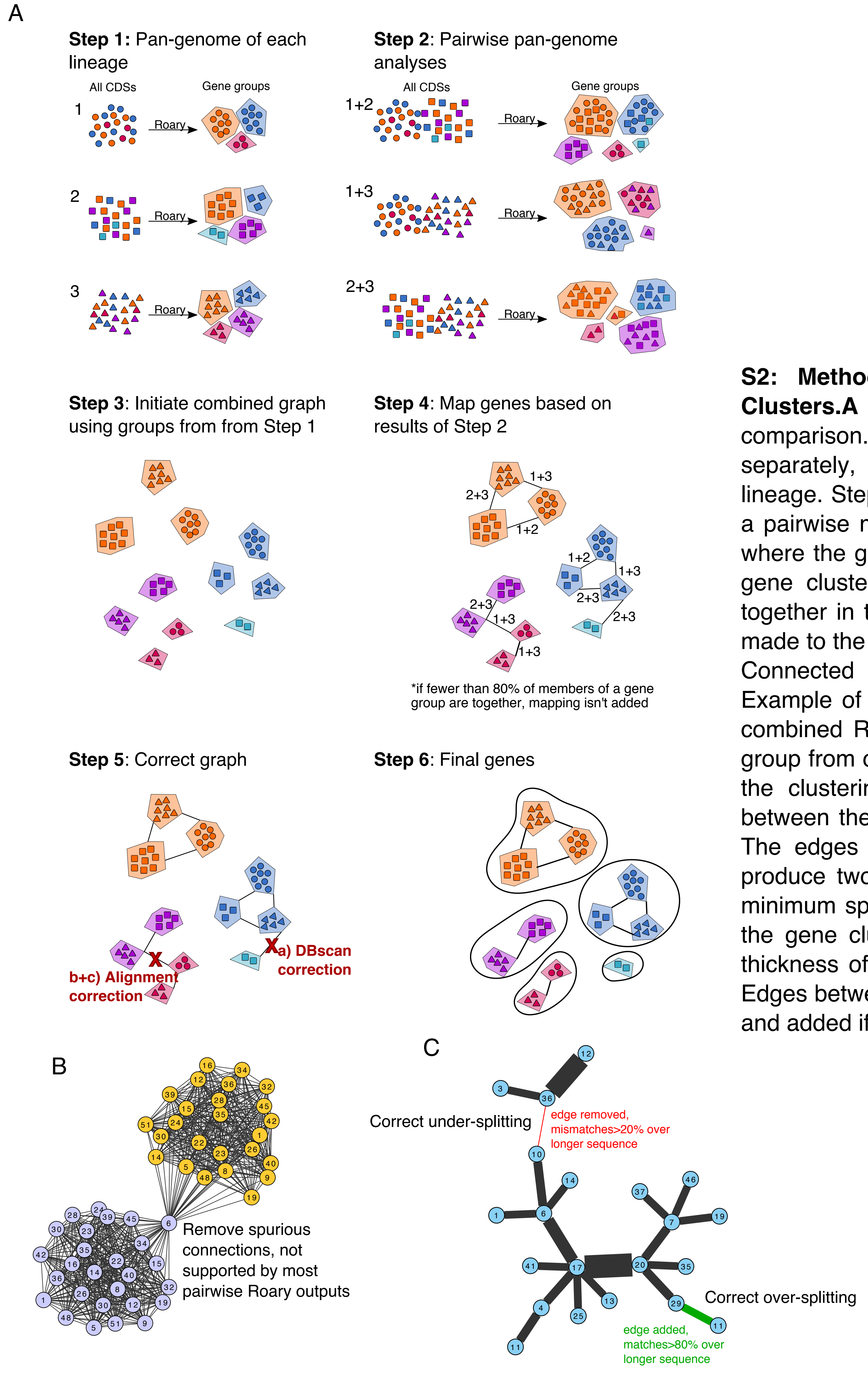

S2: Method for combining the pan-genome analysis of all PopPUNK Clusters.A Procedure for a pan-genome analysis using a pairwise Roary comparison. Step 1: a pan-genome analysis was applied on each lineage separately, generating gene clusters from all the CDSs of all genomes in that lineage. Step 2: A pan-genome analysis using Roary was applied on all lineages in a pairwise manner, generating new gene clusters. Step 3: A graph is constructed where the gene clusters from Step 1 are the nodes. Step 4: An edge between two gene clusters was added if the members of both gene clusters were grouped together in the pairwise pan-genome analysis in Step 2. Step 5: Corrections were made to the graph using density based clustering and sequence alignments. Step 6: Connected components were extracted as the final gene cluster definitions. **B** Example of density based clustering correction. The graph is a real example of a combined Roary graph as presented at the end of Step 4. Each node is a gene group from one lineage. The nodes are numbered by their Lineage and coloured by the clustering result of density based clustering. In this case, the connection between the two groups is only supported by a spurious connection of Lineage 6. The edges between Lineage 6 and the rest of yellow lineage are removed to produce two groups. **C** Example of alignment based corrections. The graph is a minimum spanning tree of the alignment between the representative sequences of the gene clusters from each lineage. Each node is a gene in one lineage. The thickness of the edge between two genes in the percent matches between them. Edges between genes are removed if they differ by more than 20% (under-splitting), and added if they match by more than 80% (over-splitting).

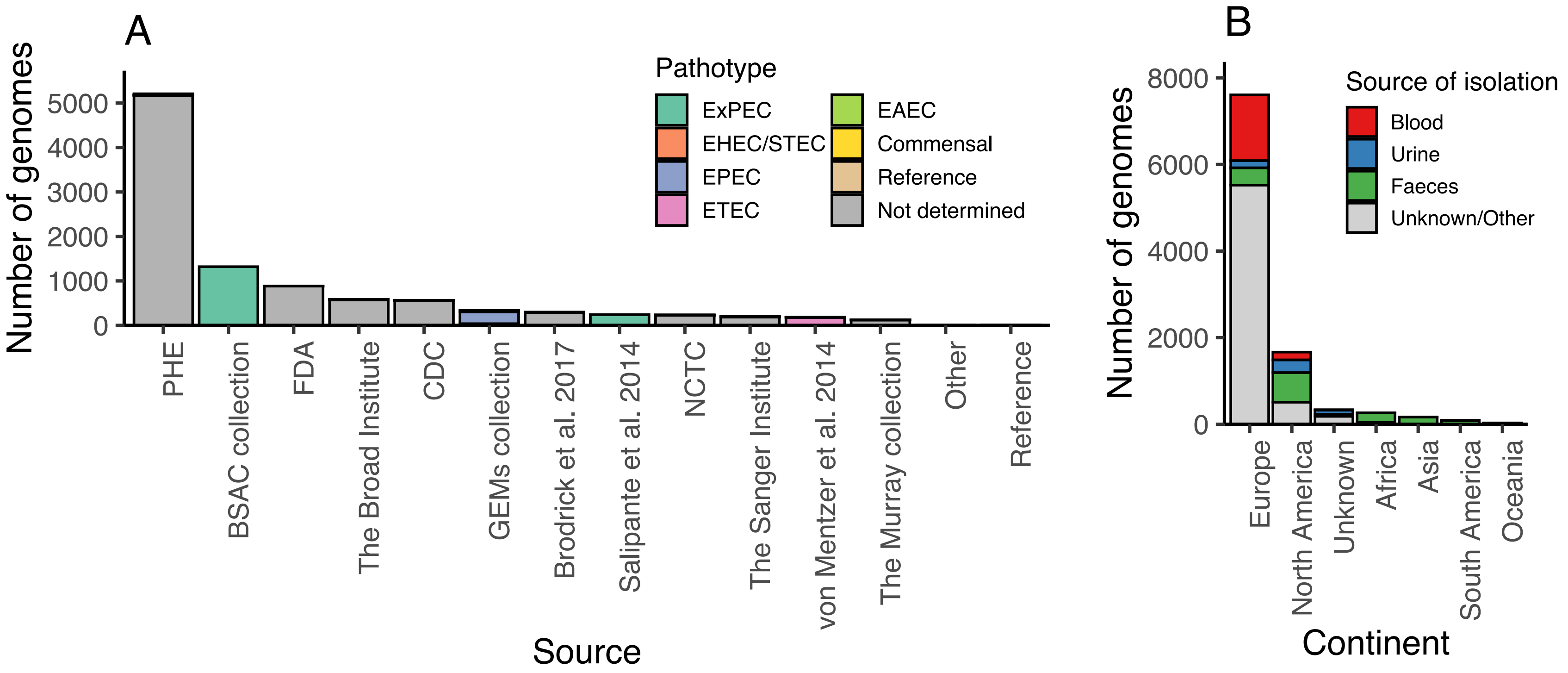

S3: Source of *E. coli* genomes. A Source of the *E. coli* genomes in the collection, coloured by the pathotype associated with the specific studies. **B** Continents from which the *E. coli* genomes were collected, coloured by source of isolation.

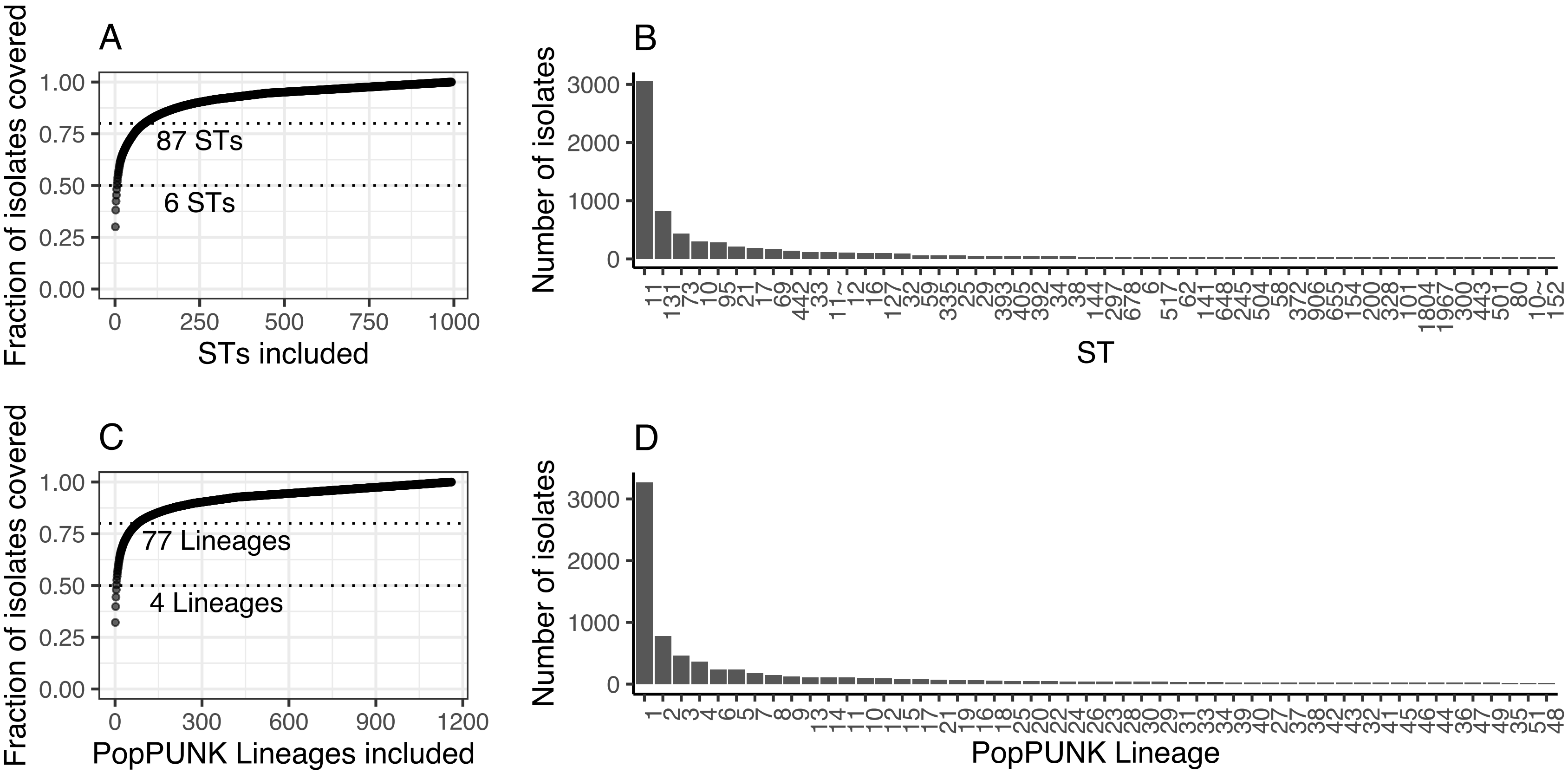

S4: Distribution of STs and PopPUNK Lineages in the collection. A,C Coverage of genome collection by increasing the number of STs (A) or PopPUNK Lineages (C) included in the study. Dotted lines: Number of STs (A) or PopPUNK Lineages (C) which accounted for 0.5 and 0.8 of all isolates in the genome collection. **B,D** Number of genomes in the fifty largest STs (B) and PopPUNK Lineages (D).

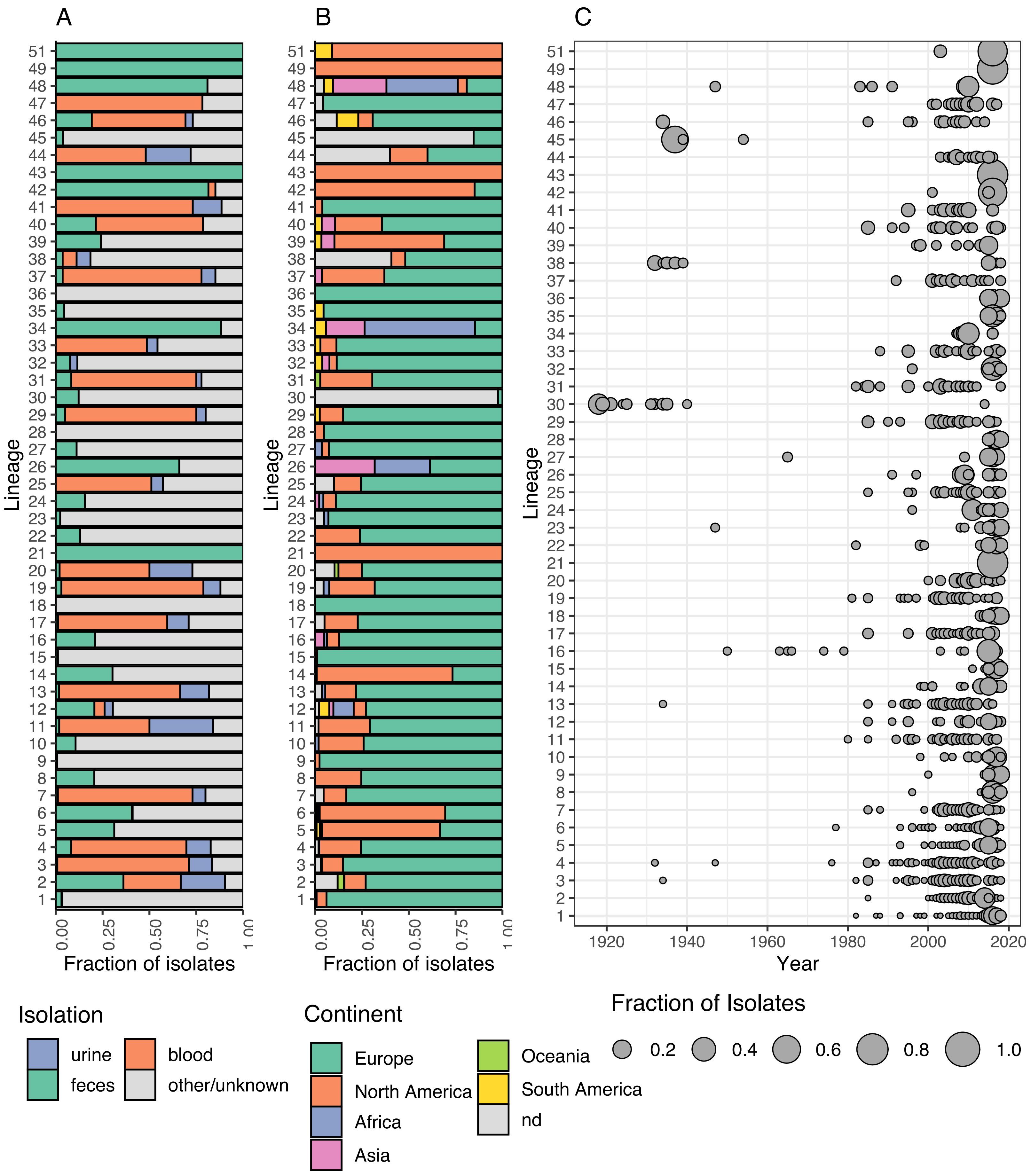

S5: Metadata associated with the lineages.A,B Source of isolation (A) and continent (B) of isolates from the fifty lineages. **C** Fraction of genomes from each of the lineages collected from each year (where metadata was available).

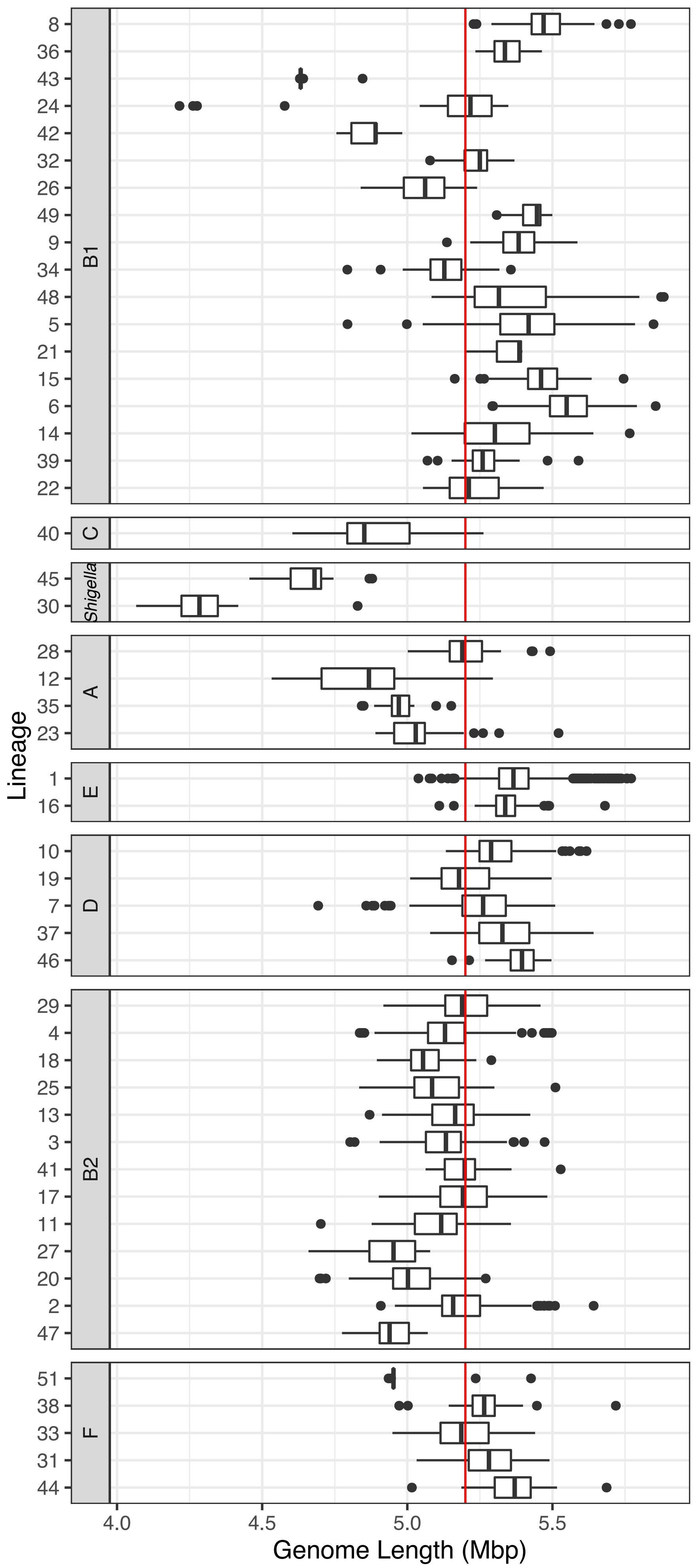

S6: Genome lengths across the lineages. Genome length, measured as total contig length, per isolate across the lineages, divided by their phylogroup. Red line: weighted-mean genome length across the entire collection.

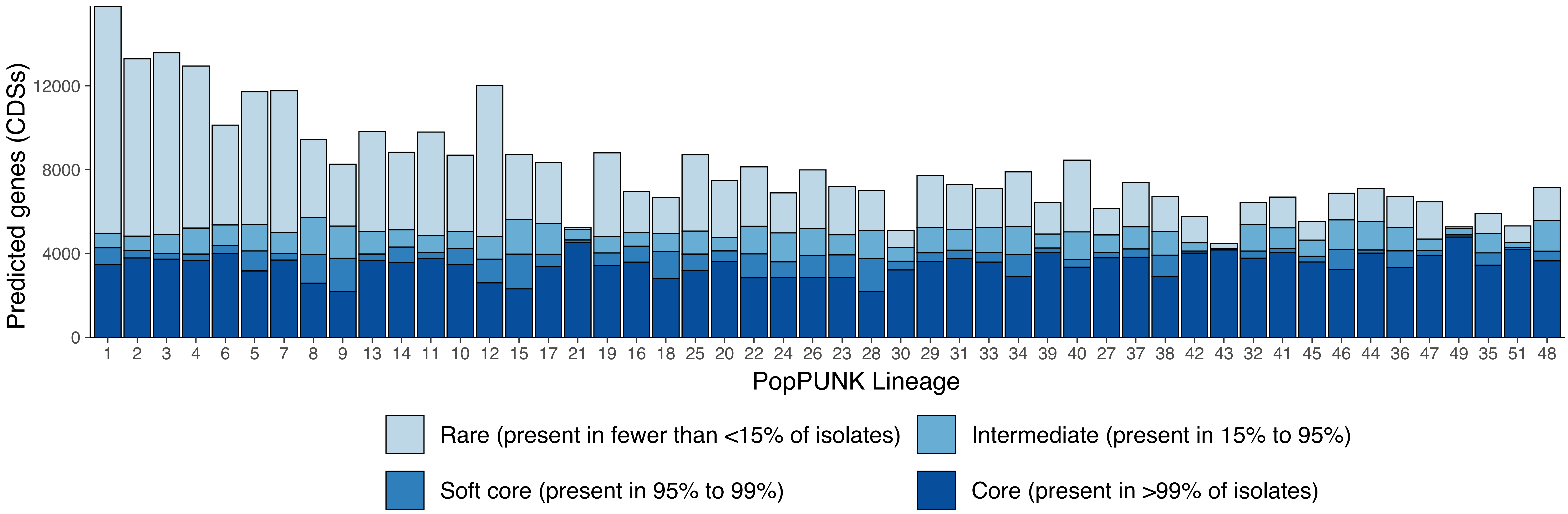

S7: Pan-genome size across the lineages. Number of predicted CDSs in each lineage in a Roary analysis per lineage, coloured by division into core, soft-core, intermediate and rare genes. Lineages 21, 43 and 49 were removed from downstream analysis due to low diversity in gene content.
